# Cardiac Amyloidosis in Venezuela: A Pending Issue

**DOI:** 10.2174/011573403X305835240715092532

**Published:** 2024-07-15

**Authors:** Juan Salazar, Mayela Bracho, Carlos Esis, Roberto Añez-Ramos

**Affiliations:** 1 Institute for the Research of Cardiovascular Diseases of the University of Zulia, Maracaibo 4004, Venezuela;; 2 Department of Endocrinology and Nutrition, Quirónsalud Hospital, Madrid 28009, Spain

**Keywords:** Cardiac amyloidosis, diagnosis, treatment, epidemiology, venezuela, misfolded proteins, pathology, cardiomyopathy

## Abstract

Cardiac amyloidosis (CA) is an infiltrative disease characterized by the deposition of misfolded proteins in cardiac interstitial tissue. Interest towards studying this pathology has been growing in the last decade, as new epidemiological insights have revealed that it is not as uncommon as previously believed. Likewise, advances in non-invasive diagnostic approaches and the identification of molecules that modify its long-term progression, even in terms of mortality, have also bolstered interest in CA. Despite this global panorama, in Venezuela, limitations remain regarding the diagnosis of CA, partly associated with a lack of knowledge of the disease. Therefore, additional efforts are necessary for clinical cardiologists to hone their diagnostic skills regarding this disease, as opportune identification is an essential step for its effective management.

## 1 INTRODUCTION

To date, approximately 42 amyloidogenic precursor proteins have been identified, of which 9 appear to be capable of accumulating in cardiac extracellular tissue. This deposition of misfolded proteins in the form of “fibrils” in cardiac interstitial tissue is known as cardiac amyloidosis (CA), whose clinical manifestations may range from asymptomatic presentations to advanced stages characterized by restrictive cardiomyopathy [1].

**Fig. (1):**
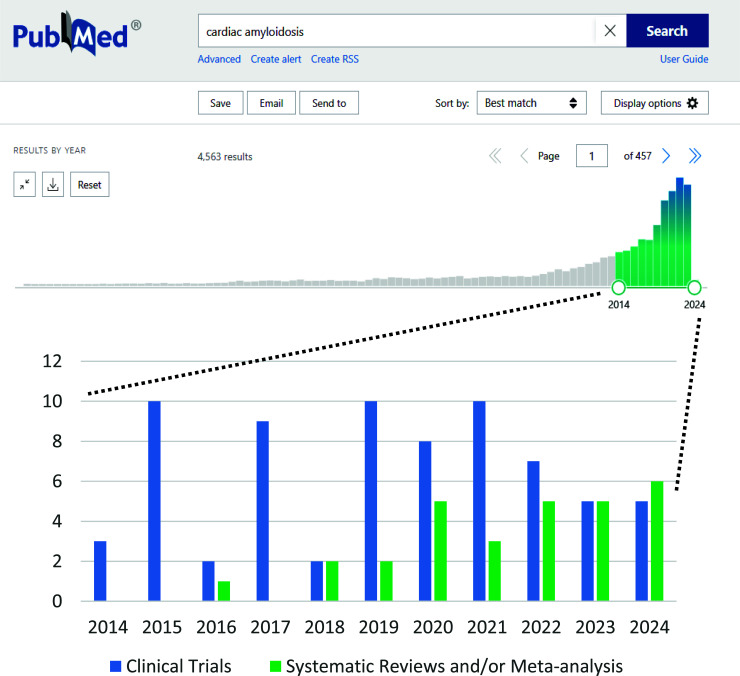
Web registry of cardiac amyloidosis publication reports in the last decade. Source: Pubmed/Medline.

Historically, CA has been considered a rare or infrequent disease, given its low prevalence in the general population. However, novel approaches to its epidemiological study in the last decade have awoken fresh interest in this topic. Indeed, several recent reports demonstrate that in the clinical course of relatively common cardiac diseases, such as heart failure with preserved ejection fraction (HFpEF), heart failure with reduced or mid-range ejection fraction (HFrEF or HFmrEF), and severe aortic stenosis, there is a frequent and widely underdiagnosed coexistence of CA [2]. In addition to this, advances in non-invasive diagnostic techniques for CA, as well as the introduction of drugs capable of reducing the morbidity and mortality associated with this disease, have attracted both clinical and research attention, with a significant number of publications since 2014 (Fig. **1**) [3].

Despite this global outlook, the deficits in CA diagnosis and reporting in Venezuela are alarming. In a thorough search carried out in the main biomedical libraries in January 2024, very few results were found directly related to any recent reports on CA in our country. There were only two relevant findings in indexed or systematized cardiology publications: The first is a pathological anatomy study by Suárez *et al.* [4], which dates back to the middle of the previous century and includes 210 cases of different types of myocardiopathy, finding deposition disorders, such as amyloidosis or hemochromatosis in 6.2% (n=13) patients. The second is a clinical case reported by a team of cardiovascular imaging specialists from the Ascardio CCR echocardiography laboratory [5].

These difficulties in diagnosing CA in Venezuela may be linked to a significant lack of knowledge of the disease. This aspect has also been evidenced in other Latin American countries [6]. Alongside this reduced awareness of CA, the limited availability of key diagnostic tools for the confirmation of CA, even in private healthcare, indirectly generates a kind of diagnostic inertia, severely limiting diagnostic possibilities. Lastly, the high costs of the necessary tests also contribute to this issue, as they cannot be afforded by the majority of the population. Furthermore, there is a notorious absence of centers specialized in CA or cardiomyopathies, which results in these specialized tests being rarely subsidized by state organizations.

Given this complex problem, we want to emphasize three aspects we consider essential to influence the timely diagnosis and treatment of this disease, particularly in the local context of Venezuela. Notably, we do not intend to specifically address the details of the approach, as there are multiple comprehensive guidelines from several sources for the management of these patients [7-9].

Firstly, we would like to highlight the importance of considering this diagnosis as a fundamental step in the identification of subjects potentially affected by CA. At this point, it should be noted that, except for cardiac magnetic resonance imaging and global longitudinal strain (echocardiography) findings, all clinical and paraclinical “red flags” can be determined in the majority of cardiac hospitalization and outpatient services in our country, simply by taking a detailed clinical history.

Regarding confirmatory diagnostic tests, both monoclonal analysis and biopsies are widely available in several healthcare centers nationwide. In contrast, access to scintigraphy with SPECT and genetic testing is much more restricted, requiring their processing through specialized centers located exclusively in our capital city. Nevertheless, this phase, although more difficult to achieve given its high cost, may not be superior to other more common cardiological procedures, such as angioplasty or pacemaker implantation.

Finally, the therapeutic approach to these patients is complex since it must encompass the use of disease-modifying drugs and specific treatment according to the underlying cardiac manifestations. The first group includes tafamidis, the only specific FDA-approved treatments for CA to date, and acoramidis, whose relatively recent discovery makes them very high-cost drugs, as well as very difficult to access even in countries with more sustainable health systems than Venezuela. However, there are international organizations that support patients with CA and facilitate the acquisition of these drugs in regions with low-income resources [10]. This panorama emphasizes the need to make an appropriate and timely diagnosis since the only way to improve the availability or access to these molecules is by correctly diagnosing these patients.

In addition to the steps summarized in Fig. (**2**), we would also like to highlight the need to create multidisciplinary teams with national and international experts in this field who could provide permanent advice on suspected cases and could serve as a link to achieve diagnostic and therapeutic objectives. In turn, this would translate into better long-term clinical outcomes.

## CONCLUSION

CA is a clinical entity that is difficult to diagnose, especially in low-income settings. Therefore, clinical suspicion should be a fundamental step in the diagnostic-therapeutic approach. A thoughtful message to avoid clinical inertia in professional practice in our country in relation to a pathology that seems more frequent every day.

## AUTHORS’ CONTRIBUTIONS

The authors confirm their contribution to the paper as follows: study conception and design: JS, MB. draft manuscript: CE, RA. All authors reviewed the results and approved the final version of the manuscript.

## Figures and Tables

**Fig. (2):**
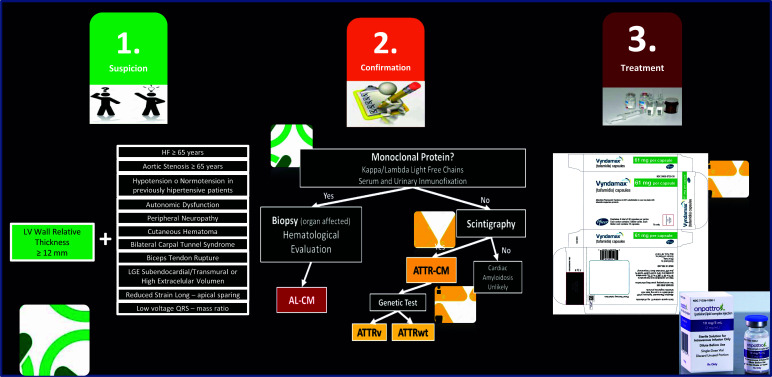
Simplified diagnostic-therapeutic algorithm for cardiac amyloidosis and its accessibility in Venezuela.

## LIST OF ABBREVIATIONS

HFpEF: Heart Failure with Preserved Ejection Fraction
CA: Cardiac Amyloidosis
